# How Health Systems ‘Software’ Factors Affect Frontline Health Workers' Humanitarian Response Efforts During Infectious Disease Outbreaks in the Rohingya Refugee Camps, Cox's Bazar, Bangladesh

**DOI:** 10.1002/hpm.70088

**Published:** 2026-05-14

**Authors:** Georgia Venner, Jennifer Palmer, Tasmia Aksi, Mahima Das Mumu, Mohammed Mirza Nu, Gillian McKay, Mahbub Ur Rahman Ujjal, Bhargavi Rao, Ahmed Hossain, Susannah Mayhew

**Affiliations:** ^1^ Department of Global Health and Development Faculty of Public Health and Policy London School of Hygiene and Tropical Medicine London UK; ^2^ Independent Consultant Bangladesh; ^3^ Independent Consultant Rohingya Refugee Camps Cox's Bazar Bangladesh; ^4^ ELRHA Cardiff UK; ^5^ Friendship NGO Dhaka Bangladesh; ^6^ Department of Anthropology Jahangirnagar University Dhaka Bangladesh; ^7^ Mansion Unit MSF London UK; ^8^ Department of Public Health North South University Dhaka Bangladesh; ^9^ College of Health Sciences University of Sharjah Sharjah UAE

**Keywords:** COVID‐19, health systems, health workers, humanitarian, outbreaks, pandemic, qualitative research, refugees

## Abstract

Humanitarian settings face a growing healthcare workforce crisis marked by staff shortages, unsafe working conditions, and limited professional development. Despite being critical intermediaries in outbreak responses, demands on health workers come at a substantial cost to their health and wellbeing. Research on health workers responding to the Rohingya refugee crisis in Cox's Bazar, Bangladesh, is limited. Focussing on health systems “software” (relationships between people and structures), this study explores health workers' interactions with different levels of governance structures, including humanitarian response governance, the health sector coordination leadership team, and their affiliated NGO management structures when responding to disease outbreaks in the camps, particularly during COVID‐19. Qualitative research consisted of 33 interviews and 4 data validation workshops with frontline health workers, and 13 key informant interviews with humanitarian stakeholders. Health workers' dependence on government camp authorities for their safety and public health enforcement produced complex relationships and sometimes compromised medical practices and professional support. Through the support of the health sector leadership team, infection prevention and control training was perceived as a key strength; however, health workers reported limited inclusion in decision‐making and being professionally unprepared during outbreaks. These experiences compounded risks to their mental and physical health, and they reported receiving minimal organisational support. These relational and structural tensions undermined worker resilience and patient‐provider trust. The findings underscore the need for outbreak preparedness and strategic reforms in humanitarian decision‐making that prioritise health workers' safety, promote inclusiveness in decision‐making, and embed sustained professional support in humanitarian health systems for future outbreaks.

## Highlights

- Health workers are critical intermediaries in outbreaks but lack protection.
- Restrictive health system structures can hinder health workers' patient care.
- Robust global health workforce policies must be adopted in crisis settings.
- Frontline health workers must be included in humanitarian sector decisions.

## 1 Introduction

### 1.1 Background

The healthcare workforce crisis, a combination of the shortage of health providers, poor working conditions, low wages, insecurity and a lack of protection, is of global concern and a significant threat to delivering essential health services worldwide [1]. Globally, there is a lack of political will and use of legal instruments to protect and improve policies to support health workers [1, 2]. The World Health Organisation (WHO) describes the health workforce crisis as one of the most critical constraints to delivering essential health services worldwide, due to poor working conditions, shortages, and insecurity [3]. In humanitarian crisis settings, frontline health workers are critical intermediaries in infectious disease outbreak responses; however, workload demands on them during outbreaks come at a substantial cost to their health, well‐being, and livelihoods [1, 4, 5, 6, 7]. Working in already strained health systems, they are commonly ill‐equipped to deal with large‐scale outbreaks, and pressure from both organisational employers and patients significantly affects their physical and mental health.

Health workers in crisis or low‐resourced settings often report a lack of PPE to keep them safe when dealing with infectious disease outbreaks [8, 9]. Research on health workers in the global south, when controlling outbreaks, shows them extending themselves beyond the response protocols and risking their lives doing nuanced and invisible work behind the job titles [5, 8, 10, 11]. For example, during the 2014‐16 West African Ebola epidemic, community trust towards treatment and testing centres was difficult to build by health workers, and they struggled to cope with chronically weak health systems, where many became infected and died [12]. Additionally, poor mental health experienced by health workers questions how far management structures will go to expose staff to risky situations, or worse, be unaware of it all happening [7, 13]. Preliminary research on how health workers are shaped by, and navigate, the health system in refugee crisis responses remains limited.

Our research focuses on the pressures affecting health workers serving in one of the world's largest refugee crises. Since 2017, around one million Rohingya refugees (referred to as Forcibly Displaced Myanmar Nationals by the Bangladesh Government) have been living in crowded refugee camps in Cox's Bazar, Bangladesh, having fled ethnic persecution in Myanmar. In a context of low pre‐existing vaccination coverage for most vaccine‐preventable diseases in Myanmar and congested living conditions in Bangladesh, camp inhabitants have been exposed to numerous outbreaks and epidemics of infectious diseases, including cholera, scabies, COVID‐19, diphtheria, mumps, and measles [14, 15, 16, 17]. The COVID‐19 pandemic response in the camps, in particular, brought hardships through movement restrictions and suspension of some key refugee livelihood, education and protection programmes, while the health system struggled to mount a rapid response to yet another new outbreak [18, 19].

Qualitative research has shown that the Rohingya community have experienced fear, discrimination, and poor treatment in non‐governmental organisation (NGO) health centres run by international and Bangladeshi charities in the camps [20, 21, 22, 23]. While most of this research was conducted during the COVID‐19 pandemic, the studies reported on some long‐standing issues in the camps which pre‐dated that pandemic, as well as harmful experiences in the Myanmar health system, including violence, extortion and discrimination under military control [24]. In the Rohingya camps, health workers are reported to experience significant stress, and are often faced with chronic shortages in resources, overcrowded facilities, minimal training and support, and threats of violence by patients; however, there is limited in‐depth research [20, 25, 26]. Overall, these reports further demonstrate the strong need to understand the scale of the humanitarian crisis frontline health workers are trying to respond to, especially in the face of widespread distrust and an overstretched health system.

#### 1.1.1 Health Systems “Software” as an Analytical Lens Guiding Framework

Applying a health systems framework, which considers software and hardware elements of a health system [27], provided a useful analytical lens to explore health workers' experiences in the specific social and political context of the Rohingya refugee camps during infectious disease outbreaks. Systems hardware refers to functional aspects of a health system, and is commonly compared to WHO's health system blocks, that is leadership and governance structures, human resources, finance, medicine and technology, infrastructure, and information services [27, 28]. Hardware aspects have been the primary focus of disease outbreak responses in the Rohingya camps, with initiatives prioritising disease monitoring and infrastructure development, and in wider health interventions where donors are often driven by hardware investments and short‐term needs [27, 29, 30]. Systems software, on the other hand, is concerned with non‐linear, human‐centred relationships, values, and norms between people and structures within the health system [27]. This framework has been applied in previous health systems scholarship, such as in understanding how social and political factors shape, enable, or constrain health system responses to HIV, sexual and reproductive health services, community health worker performance, and TB research in resource‐limited settings [31, 32, 33].

We applied a health systems software lens in our analysis to conceptualise how feelings of being valued, interpersonal relationships, and experiences of power were shaped by health workers' interactions with different levels of governance structures during outbreak responses in the camps [33]. In this study, governance structures are defined as higher‐level decision‐making bodies that have direct or indirect authority over health workers, including the humanitarian governance level run by Bangladesh government authorities known as the Camp‐in‐Charge (CiC); the health sector coordination leadership team overseen by (WHO); and the health workers' affiliated organisational management structures, including senior leadership overseeing their role [33]. Specifically, we analysed how the relationships between these elements enabled or constrained health workers' care practices and motivations to work in the Rohingya humanitarian response.

## 2 Methods

### 2.1 Study Setting

The Rohingya are the largest stateless group in the world and have suffered decades of persecution, most notably the 2017 indiscriminate attacks from the Myanmar army, causing an influx of about 711,369 refugees into Cox's Bazar's coastal region of Bangladesh and other bordering countries, with an estimated 10,000 Rohingya killed [34, 35]. Currently, 968,981 Rohingya refugees live within Cox's Bazar District, and 36,539 on the island of Bhasan Char, totalling just over 1 million registered refugees. There are 33 mainland camps in Cox's Bazar District within Ukhiya and Teknaf Upazilas [36].

The response in Cox's Bazar District is overseen by various government entities, notably the Refugee Relief and Repatriation Commissioner (RRRC), and they work with the international humanitarian response overseen by the Inter‐Sector Coordination Group (ISCG) led by the UN Principle Coordinator [37]. The health sector is jointly led by the Ministry of Health and Family Welfare/Civil Surgeon (MoHFW/CS) and WHO, and is responsible for ensuring adequate training and resources are provided to health workers by NGOs contracted to deliver health programmes in the camps [38].

At the time of data collection (2022–23), health facilities were run by approximately 80 different health implementing partners from international non‐government organisations (INGOs) and national NGOs. Secondary and tertiary national care centres in the surrounding Ukhiya and Cox's Bazar district also served the camp settlements. The majority of frontline clinical staff are Bangladeshi, while Rohingya health workers are limited to serving as community health workers. Although Rohingya refugees can informally work as community health workers (CHWs) in Bangladesh with a small stipend, there have been limited opportunities for them to work formally and in clinical spaces; this is thought to have contributed to the perceived low quality of care in clinics reported by Rohingya patients [21, 23]. Care‐seeking from traditional healers and local shops selling medicine within the Rohingya community is thus often a preferred option for many camp residents, despite it being costly [24].

Government authorities in charge of camp administration are known as the Camp‐in‐Charge (CiC) offices. They oversee all sector activities, camp residents and commonly engage with health centres and operations. Civil servants staff CiC offices, and some offices have a designated health coordinator who liaises with health clinics. CiC offices wield significant authority over camp governance, overseeing the activities of the mahjis (Rohingya community leaders appointed by the Bangladesh Government), imams (religious leaders), refugee communities and NGO workers, including health workers, operating in the camps.

### 2.2 Study Design

This qualitative study consisted of a collaboration between the London School of Hygiene & Tropical Medicine (LSHTM) in the UK, North South University (NSU) in Dhaka, Bangladesh and Friendship Bangladesh, a Social Purpose Organisation involved in the humanitarian health response, including staffing and overseeing services across various health facilities in the camps. Partner details and further study design details are found in Supporting Information S1. It was conducted between October 2022 and July 2023 and drew on interviews with 33 facility‐based Bangladeshi health workers, 13 humanitarian actors involved in health programming for the Rohingya refugee response in Cox's Bazar, and 4 data validation workshops with a second set of Bangladeshi health workers. We attempted to enquire about various outbreak experiences since 2017, but COVID‐19 had strong reflection points, being the pandemic at the time, and the fact that there was a high turnover of staff meant previous outbreaks were less frequently alluded to. Perspectives of community health workers, including Rohingya, and the relationships between patients and providers in the response, are explored separately (forthcoming).

### 2.3 Data Collection

#### 2.3.1 Interviews

Health workers participating in in‐depth interviews (IDIs) came from four international and three Bangladeshi NGOs responsible for service delivery in primary health care centres, health posts, and specialised health facilities (such as isolation and treatment centres [ITCs]) in at least 17 camps. Some informants did not record their camp number, while others worked across several camps. Outlined in Table 1, participants included clinical managers, clinical officers, medical assistants, nurses, midwives, dental assistants, mental health workers, and public health promoters (an explanation of professional cadre definitions can be found in Supporting Information S2). Health workers were recruited through study research partners' networks, organisations listed in the health sector's publicly available electronic database, sharing study information with health organisations during health sector meetings and snowball sampling. Newly recruited health workers with minimal experience working in the camps were excluded from the study as they were less likely to provide in‐depth reflections on their experiences responding to outbreaks. Semi‐structured topic guides for IDIs centred around health workers' career journeys working in the camps, experiences of infectious disease outbreaks, training and professional development experiences, camp environment and safety, relationships with patients and communities, and recommendations for future support.

**TABLE 1:** Summary of data collection methods included and total participants.

Data collection type	Number	Ethnic background	Gender	Professional background^a^	Type of organisations
In‐depth interviews (IDIs)	33	Bangladeshi	13 females 20 males	Clinical managers (trained medical doctors); clinical officers (trained medical doctors); medical assistants (MA)/MA supervisors; nurses/nurse supervisors; midwives; public health promoters; mental health activity managers; dental assistants	International and bangladeshi NGOs
Data validation workshops (WS)	4	Bangladeshi	9 females 11 males	Workshop 1: Clinical managers (trained medical doctors); Workshop 2: Clinical officers (trained medical doctors) Workshop 3: MA/MA supervisors Workshop 4: Nurses/nurse supervisors and midwives	International and bangladeshi NGOs
Key informant interviews (KIIs)	13	International and bangladeshi	5 females 8 males	Humanitarian and technical experts working in Cox's bazar, high‐level decision‐makers within the humanitarian health sector	International and bangladeshi NGOs, inter‐governmental organisations and funders
Total participants	63 (3 health workers participated in both an IDI and a workshop)				

^a^ Some participants had changed positions between the time they were working in key outbreaks discussed during the interview, and were able to share information from multiple perspectives. Participants are classified according to the main perspective shared.

Humanitarian actors participating in key informant interviews (KIIs) were recruited through information in the Health Sector Coordination Team (HSCT) database, purposively recruited using professional networks across the health facilities, and snowball sampling. The KIIs captured higher‐level system and organisational perspectives from humanitarian stakeholders involved in the health sector. Topic guides for KIIs centred on humanitarian actors' roles and decision‐making experiences responding to infectious disease outbreaks in the camps, comparing responses, challenges and successes in outbreak and health responses, training and support structures for health workers, and recommendations.

Interviews took place in English or Bengali (translated in real‐time) and in private locations of the participant's choice within or near the camps, or remotely via Zoom. Interviews were recorded with consent, and for informants who did not want to be recorded, detailed field notes were taken.

#### 2.3.2 Data Validation Workshops

Four data validation workshops, with 20 participants in total, were held with health workers divided into similar professional backgrounds (see Table 1) from at least 12 camps. The workshops were conducted during a second research visit to gather feedback on preliminary findings and to inform the development of recommendations for the health sector. The majority of workshop participants were not interviewed previously and were recruited through emailing health organisations and research partners' support. Workshops 1 and 2 were held in English, while workshops 3 and 4 were held in Bengali. Workshops were held in a private meeting space in Cox's Bazar city, each lasting four hours, and were audio recorded to produce transcripts.

#### 2.3.3 Analysis

Using the topic guide and research themes, preliminary findings and emerging themes from interviews of the first data collection visit were mapped out by members of the field team (GV and TA). GV then carried out a rapid thematic analysis of data from about half of the IDI transcripts to produce preliminary findings and group early themes to assist data validation workshops.

Following the workshops and the end of data collection from the second research visit, themes were mapped out again with the field team (GV, TA, MDM, MMZ). GV then undertook an in‐depth analysis following Braun and Clarke's [39] six‐phase approach, interpreting the data reflexively using both inductive and deductive methods. Themes were informed by the outcomes of the data validation workshops and the topic guides, while inductive coding enabled the identification of nuanced findings. To enhance theoretical coherence, Sheikh et al.‘s health systems framework exploring hardware‐software conceptualisation was then used deductively to guide the analysis, including the organisation of codes and mapping themes aligned with the identified software components of the system (with a focus on relationships and power) against systems hardware (humanitarian response governance, health sector coordination leadership, and organisational management) [27]. The results were organised into different levels of response governance structure (an aspect of hardware), with software‐related themes explored within each level. Applying a hybrid analytical strategy with thematic analysis and framework analysis provided the flexibility needed to capture detailed experiences of health workers, and a structured lens to refine themes and assist in mapping across different levels of the humanitarian health system response. While we primarily used this framework as an analytical lens to help interpret the data, and we did not examine software elements such as values and norms in substantial depth, the hardware–software framework was used as a tool to situate the various influences shaping health workers' experiences within the health system.

Theoretical saturation was reached after detailed coding in NVivo12 of 22 (of 33) IDIs, and 2 (of 4) data validation workshops. A rapid analysis of the remaining data, 11 (of 33) IDIs, and 2 (of 4) workshops was manually completed to ensure consistency of findings and identify any deviations from our main themes. All KIIs were rapidly analysed manually to support or challenge the themes identified. Further information regarding data collection and analysis can be found in Supporting Information S1.

#### 2.3.4 Strengths and Limitations

In terms of strengths, a large number of interviews, KIIs, and data validation workshops were conducted over the year of data collection. As such, this qualitative study captured a diversity of perspectives from a range of health providers working for different organisations in the camps. Participants provided a rich description of experiences, particularly of the recent COVID‐19 pandemic; however, high staff turnover meant fewer participants were able to talk about the pre‐COVID‐19 outbreaks. Keeping the participants' identities and professional affiliations completely anonymous mitigated hesitations to participate and allowed for in‐depth accounts to be shared with the research team.

In terms of limitations, the humanitarian health response spans over 80 different implementing health partners with over 1000 health workers; as such, this study only provides a snapshot of experiences. However, the majority of experiences analysed were shared across providers. There is the possibility of response bias, where some health workers may have felt constrained in what they could share due to concerns about professional repercussions. Additionally, our study did not include perspectives from Rohingya refugees to triangulate findings at the time of data collection due to feasibility, time, funding and scope of the research project; however, there was extensive literature available on this topic.

The multilingual nature of interviews raises the potential for missed translation or misinterpretation of responses by the translator. As the analysis was primarily conducted by one researcher (GV) from a western setting and as part of her PhD, there remains a possibility of individual interpretive bias; this was mitigated through a collaborative and reflexive approach when formulating the results and analysis, such as triangulating emerging themes with co‐authors, including research assistants and partners with Bangladeshi and Rohingya origin, for contextual accuracy and credibility. Additionally, the data validation workshops were conducted to both confirm and challenge the preliminary findings identified by GV. Data from CHWs (both national and Rohingya) were also excluded as they had different experiences, but will be published elsewhere (forthcoming).

## 3 Ethics

This study has been approved by the LSHTM Ethics Committee (reference: 28130) and the NSU Institutional Review Board (reference: 2022/OR‐NSU/IRB/0809). Local permission for camp access for interviews was granted by the Office of the Refugee Relief and Repatriation Commissioner. All participants provided informed consent (written or verbally recorded) in their preferred language. To protect the confidentiality of health workers and their associated organisations, identifiable features such as organisation names and camp locations are not included in this study.

## 4 Results

**FIGURE 1:**
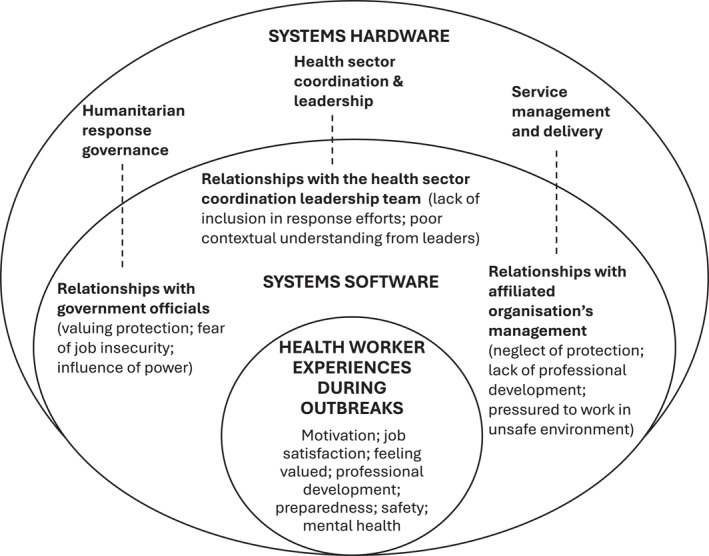
Health systems software interactions shaping health worker experiences during outbreaks in Cox's Bazar.

The following sections explore health workers' reported feelings of being valued, experiences of providing care, interpersonal relationships, and power influences that have shaped their interactions with different levels of governance structures during outbreak responses in the Rohingya refugee camps. Findings are conceptualised in Figure 1. Discussions centred on how systems functioned and how services were delivered during infectious disease outbreaks from 2017 (influx of refugees into Cox's Bazar) to 2023 (end of data collection). The COVID‐19 pandemic at the time (2020–23) and a scabies outbreak (2023) were the main outbreaks reflected on by health workers and humanitarian informants, given their recency.

### 4.1 Humanitarian Response Governance and the Pivotal Role of the Camp‐In‐Charge (CiC)

#### 4.1.1 Valuing Protection From Government Officials

Civil servants working under the Bangladesh Government serve as the CiCs in each camp, and have authoritative oversight during disease outbreaks was felt by most health workers interviewed to be critically important, particularly during the COVID‐19 pandemic, where efforts focused on containing and mitigating the spread of illnesses. CiCs worked through the mahjis (government‐elected Rohingya community leaders) to enforce specific public health measures. Health workers generally perceived the involvement of CiCs and mahjis in this work positively.

CiC and mahji interactions were perceived to be particularly useful to ensure people who tested COVID‐19 positive respected the 14‐day isolation periods, which they were required to spend in ITCs:

So everybody [was] hiding their [COVID‐19] signs, symptoms, everything. Then I talked with the CiC […] He's a very good person […] he started communication with mahjis and camp leaders [saying] that if anyone face[s] any kind of health problems, like coughing, fever, and anything related to COVID[‐19] they should inform the proper person and they should be in the COVID Hospital.

(KII_P03_Health Manager for INGO)

However, the CiCs' practices in outbreak response were perceived to be coercive, where they not only enforced power inequities between refugees and authorities but also shaped health workers' clinical decision‐making and further eroded patient trust in both the health system and its frontline providers. For example, participants described several ways in which they enforced public health behaviours under government guidance during the pandemic, such as by forcefully admitting people to isolation centres or threatening people's access to food for leaving isolation:

We let them [patients] know if you leave the facility [a COVID‐19 ITC], we will let the Majhi know, and your [food] ration will not be given to you in the future. Or your [food ration] card will be seized. We must, because some critical patients are not concerned about their physical condition.

(WS 01 Participant_Clinical Managers)

Some health workers appeared complicit in these enforcement measures, while others resisted them. For example, during the pandemic, when routine vaccination uptake declined, a CiC representative wanted to threaten food rations to increase uptake, but a public health worker resisted:

One of the CiCs, he was telling [me], when the [routine] vaccination [started] for [people aged] 12–18 years, he was saying ‘just stop the food’ [if they do not receive the vaccination]. Like we can't do that. […] we cannot force anyone to take vaccination. It should be voluntary.

(KII_P08_Public Health Worker)

#### 4.1.2 Fear of Job Security Under the Power of the CiCs

When health workers faced conflicts with patients, they often felt CiCs were the only types of authority capable of protecting them and resolving issues in the camps, sometimes even being more relevant than managers in their organisations. This highlights how relational dynamics and trust with camp authorities, key ‘software’ elements of the system, directly shaped health workers' sense of support. For example, one health worker expressed feeling neglected during camp visits from their organisation's international funder, as these visits often seemed to prioritise feedback from refugees over theirs. The CiC in their camp, on the other hand, often had a full picture of the pressures health workers faced:

CIC sir is friendly [… he] listens to their [the Rohingya community’s] problems, but yeah, he knows the actual scenario. But our donor does not want to listen to our problems, so the CiC supports us so much.

(IDI_P34_Clinical Manager)

However, the CICs' support was unpredictable, and their reinforcement of power created an environment of uncertainty for health workers, weakening trust in this governance structure meant to protect them. The CiCs' support could also be unpredictable. For example, the CiCs were also accountable to the refugee populations and sometimes sided with them during conflicts between health workers and patients. A health worker shared their hesitations about seeking assistance from CiCs when dealing with difficult patients due to fear of professional disciplinary actions:

Sometimes the patients get aggressive […] if we want to take any action, then we have to go to the CiC and other officers. It’s a long procedure. Sometimes they do not even take the complaints. But if we do anything, for example, tell them [the patients] anything with a loud voice, the authority [the CiC] takes immediate action against us.

(IDI_P05_Midwife)

Additionally, CiCs had substantial control over clinical operations during the COVID‐19 pandemic, and these pressures forced health workers to adjust constantly, sometimes putting them at risk. A clinic serving a remote population in the camp at one point had many health workers infected with COVID‐19, reducing their numbers from 30 to 5 available staff. The centre had to stay open as it served a population of 40,000, causing huge pressure:

[This] means one doctor was working around the clock, because … we have to have services running 24/7 […] you can imagine the pressure that puts on five people. We did have that conversation with the CiC […]. And they're like, ‘No, you have to [stay open]’.

(IDI_P01_Clinical Manager)

### 4.2 Health Sector Coordination and Leadership Team

#### 4.2.1 Feeling Included to Build Infection Prevention and Control

In the camps, the dynamics and relationships between frontline providers and the health sector leadership team were complex. To some health workers, mainly those at a senior level, WHO and UN coordination efforts were regarded as being inclusive, with one informant saying:

We are coming together as a humanitarian response family to overcome these situations.

(WS 01 Participant_Clinical Managers)

Infection prevention and control (IPC) practices in the camps evolved with each new outbreak; however, there were no response‐wide IPC guidelines and training until the COVID‐19 pandemic. These efforts, led by WHO IPC specialists, were seen as transformative by health workers in regard to their professional development. They included activities such as implementing IPC score cards for clinics and training IPC Focal Points within health organisations, and were largely sustained after the pandemic subsided.

One of the best things that we've gotten out of COVID is an infection prevention and control committee and an efficient body that works just for infection prevention and control. That is, I think, the biggest achievement out of Cox's Bazar at the moment.

(IDI_P01_Clinical Manager)

The health sector‐led IPC initiative had positive impacts on health workers' professional development as well as their relationships with programme managers. The investment in professional development and a sense of purpose around the importance of IPC strengthened health workers' capacity to adopt and maintain IPC practices. For example, a clinical officer working as an IPC focal person for their organisation shared their experiences during the pandemic.

I was involved in the planning and the construction of the ITC [isolation and treatment centre]. Our programme director at that time, [they] wanted it to be built in a way that would be able to contain the disease and our staff would not get infected from the patients[…]He sent us for the [IPC] training. And then we actually gave guidance on how it should be constructed.

(IDI_P31_Clinical Officer and IPC Focal Point)

#### 4.2.2 Forced to Adapt to Wider Coordination Challenges

Apart from these popular IPC efforts, however, health workers mainly characterised COVID‐19 response decisions as chaotic and reported feeling overburdened in managing outbreaks. These forced adaptations and other challenges, compounded by resource shortages, left facility‐based health workers shouldering much of the responsibility of patient tracking, counselling, and trust‐building to ensure communities were following government rules for COVID‐19. These frustrations reflected deeper software factors, including poor communication and limited consultation with frontline staff, which led to excessive efforts beyond their clinical duties. Most health workers offered detailed critiques of nearly every element of COVID‐19 patient management:

When a patient comes to us, we suspect COVID, we ask them to give a sample… If they don't, we include our HP [health promoter] here…We send the sample to Cox’s Bazar lab…This process took three to five days… And it was too challenging for those people [the health workers] to try to convince the patient [to go back to ITC].

(IDI_P26_Medical Assistant)

Descriptions of the challenges health workers faced in COVID‐19 were quite similar to those related to earlier outbreaks, demonstrating how unprepared the health system still was in early 2020 for achieving outbreak control:

I witnessed the outbreak of cholera. At that time the patient flow was huge so we had to take on a lot more pressure […] The diphtheria outbreak was same, there were too many patients.

(IDI_P02_Medical Assistant)

After the COVID‐19 pandemic subsided, a large outbreak of scabies in 2023 continued to add pressure on health workers. A health worker explained the stress of distributing scabies medication and the repercussions of patients taking excess medicines in a context of limited resources:

Now there is a scabies outbreak[…] Sometimes it happen[ed] that they [the patients] are taking medicine from our facility in the morning, in the evening, they go to another facility and took another kind of medicine[…] this is not only reducing our limit of stock, but also it is creating resistance [anger] among the patients.

(WS 02 Participant_Clinical Officers)

Despite health workers reportedly sending alerts about a scabies outbreak, the health sector's response was considered slow. This disconnect points to reflected weak coordination practices within the health sector leadership, and affected trust among health workers who felt ignored when raising concerns:

As far as I know, the other actors [health organisations in the camps] are not doing that much for scabies, if they have done, the scenario could be different nowadays, the number [of cases] could be lower.

(IDI_P26_Medical Assistant)

#### 4.2.3 Lack of Relationship With and Contextual Understanding of Decision‐Makers

The perceived disconnect in NGO leadership not only reproduced structural inequities between decision‐makers and health workers in the frontline, but also undermined health workers' confidence in the guidance provided and weakened trust in the coordination system. The health sector's leadership response operated out of Cox's Bazar town, while the refugee camps were located up to 3 hours away by road. This resulted in health workers feeling isolated, preventing them from providing comprehensive feedback to the higher decision‐making authorities in the health sector about their day‐to‐day realities:

It's so much easier to talk about response and everything and do this and that when you're [the decision‐makers] sitting up there [in Cox’s Bazar]. We're the ones running around the fields in heat, rain, storm, winter and everything […] But we have to remain silent.

(WS 01 Participant_Clinical Managers)

Given these barriers, health coordination efforts were perceived by health workers to be led by individuals lacking contextual experience in the refugee camps, leading to frustrations and health workers questioning the credibility of advice from sector leaders. It reinforced power imbalances, which not only fuelled frustration but weakened trust in sector guidance and constrained health workers' ability to provide contextually relevant care.

They [the sector leaders] are coming [to the camps] at 10 [am]. And they're leaving at 12 [pm]. What are they doing in two hours? […] The health sector could be more effective if they are not sitting in cities[…] whenever we go into any engagement, like any training sessions, we always try to like show the real situations[…], but I don't know if they have been taken seriously or not.

(IDI_P27_Medical Assistant)

### 4.3 Service Management and Delivery in NGO‐Run Facilities

#### 4.3.1 Feeling Neglected in Training and Preparedness for Health Workers During Outbreaks

For COVID‐19, health workers reported an abundance of short training sessions offered by national and international experts. However, these trainings tended to only be available when an outbreak was occurring, rather than for anticipatory outbreaks. This reflected deeper software challenges, including a perceived lack of prioritisation of preparedness training for health workers. Health workers also questioned why they underwent intense training for anticipatory crises like cyclones and fire in the camps, yet these efforts did not extend to new disease outbreaks.

I think that one will be my biggest feedback because we need to do some practical drilling, for preparations of any response that we experience […] so many countries are prepared, like in Africa, they had the Ebola response [before the COVID‐19 pandemic]. So then they knew, but for us, it was totally new.

(KII_P05_Public Health Worker)

In addition, the rapidly changing guidelines made it nearly impossible to institutionalise learnings before guidance changed again. Frequent updates, along with high staff turnover, required constant retraining, straining time and resources and were thought, by health workers, to result in harmful consequences for the patient nor helpful to the context they were working in, stating, *“sometimes we have to set aside the guidelines. Just for the sake of the patient, to save the patient.”* (Workshop 01 Participant_ Clinical manager).

Like many health workforces globally, health workers in the camps struggled to secure adequate PPE against COVID‐19. Some health workers reported buying protective equipment with personal funds.

In the first two months of COVID we were provided with masks, with only one layer, at first we started complaining, some were revolting, but I mean we are healthcare providers, we cannot step back, whatever we have, whatever we could afford by ourselves, we actually had to buy PPE, even goggles and face shields from our own money.

(WS 01 Participant_ Clinical Manager)

Even when PPE was available, early on in the pandemic, some health workers were advised not to wear masks as their organisation was cautious of patients being fearful of seeking care if needed. This highlighted software elements related to feeling devalued as a health worker, where patients' needs were prioritised over their safety.

I was discouraged [from management] to wear it[…] the Rohingya population will think the disease is already in the camp […] then the [organisation] decided to take COVID‐19 patients who are mildly symptomatic, they [advised us] to do the test, but without PPE how can I collect samples [safely] from a symptomatic patient?

(WS 02 Participant_ Clinical Officers)

Despite all these challenges, the majority of health workers were determined to care for their patients, even in the face of infection risks to themselves.

I am more concerned about how I can heal the patient […] this is my responsibility. I only focus on how I can make that patient get recovered […] I do not think much about myself.

(WS 04 Participant_Nurses and Midwives)

#### 4.3.2 Coping With Care Demands and Unsafe Working Conditions

The relationship between health workers and their managers was influenced by the support available for their mental health and wellbeing. Feeling overworked was a common experience for health workers in the camps and added to the challenges of maintaining healthy livelihoods.

I can say that when a pandemic or an outbreak occurs, our health workers need to work more time without any rest. And this affects mental as well as physical well‐being.

(IDI_P26_Medical Assistant)

During the pandemic, health workers had to endure daily staff shortages and pressures to keep their clinics running, forcing them to make difficult decisions. Chronic understaffing during the pandemic exposed software challenges, where it not only intensified their stress but also exposed how organisational management structures placed disproportionate responsibility on health workers and affected their mental health and wellbeing. One clinical manager reflected on a tragic story of a baby dying in their mother's arms while waiting in line. The manager was the only doctor available in an ITC facility at the time:

When it was pandemic time, I was the only medical doctor in the ITC facility […] And when I was coming to the last patient, it was a baby. And the baby was dying already. And I was mentally too broken that [if] I could have come to this patient […] first, then maybe [they] could survive. And I was mentally traumatised for several weeks.

(WS 01 Participant_Clinical Managers)

Clinical managers were often forced to make difficult decisions and go above and beyond their official responsibilities to keep their staff working and safe, even when understaffed. For example, some health workers were evicted from their homes by landlords due to stigma and fear of spreading the virus, and a manager described inviting staff to stay in their homes.

You're also being overloaded with responsibilities that go well above and beyond what you're supposed to do […] our staff being actively evicted, it's not my problem as the facility manager…[but] in a pandemic situation where we were isolated[…] we do go above and beyond to support them in any way, invite them in our own homes.

(IDI_P01_Clinical Manager)

A significant amount of funding and resources went into building ITCs for COVID‐19‐infected patients in the camps. Many health workers stated that running ITC centres and ensuring they were filled with patients and that the patients did not escape or choose to leave, created large pressures on them.

When we started our [ITC] facility for a few days, we [were] not getting any patients [due to fear of ITCs]. […]there was huge pressure and pressure from the management that you need to have patients.

(KII_P03_Health Manager for INGO)

Health workers also had to endure incredibly high temperatures in the ITCs, as some were built as tents with low temperature‐regulating capabilities. One clinical manager, fearful of their staff's risk of overheating, tried to move their staff, but the NGO's local management refused. The manager then sought assistance from their international management but was later let go from their role for overstepping in the organisation's hierarchy. These rigid organisational power dynamics impeded health workers from speaking up, raised fears of job loss, and limited their ability to work in safe environments.

Finally, organisational management structures, compounded with a poorly funded humanitarian response, contributed to unstable hiring structures for frontline workers who were vulnerable to short‐term contracts and job insecurity in the camps, stating, *I am trying to treat this patient, but I don't even know if I will have a contract next week*. (WS 02 Participant_Clinical Officers).

## 5 Discussion

Our research provides new insights into understanding the experiences of health workers and the dynamics of their relationships to governance structures in the Rohingya refugee response in Cox's Bazar. Exploring software elements interacting with hardware elements of the humanitarian health system and their effects on frontline health workers revealed important issues with government authorities, the health sector leadership team, and NGO management structures intended to support health workers. We found that frontline health workers perceive a need for an authoritative presence to ensure their safety and to mitigate disease spread in the camps. They felt unsupported in certain public health enforcement strategies and programming decisions that risked compromising ethical, humanitarian patient care. Despite significant support in IPC, health workers felt unprepared for new outbreaks and unsupported by high‐level decision‐makers in the health sector. Health workers felt their employing organisations did not understand their clinical realities and perceived an overall lack of supportive day‐to‐day management and prioritisation of patients' needs. Challenges experienced at each governance level compounded one another, so that, overall, health workers felt their safety and needs fell well below those of patients.

Sheikh et al.‘s [27] health systems software‐hardware framework offered a useful and nuanced structure to map various structural and relational influences that shape health workers' professional ecosystems during outbreaks [30]. Similarly applied by Kok et al. [33] they conceptualised community health worker (CHW) performance through the interactions between hardware and software factors; for example, if CHWs had weak relationships with the community, targeted interventions within hardware levels, such as training, could be introduced to strengthen these relational dynamics. However, the authors stress that interventions at the hardware level should not replace software interventions, and both are needed. Our findings also suggest that structural interventions, such as formalising health worker participation in decision‐making structures and implementing WHO safety recommendations, are required. In addition, software interventions within the health sector leadership team, such as relationship building with health workers, are needed. Overall, using the framework has limitations, notably that the term “health system software” is broad and open‐ended, and there is a limited body of research applying the concept to guide accurate implementation [27, 30]. Additionally, there is limited research applying Sheikh's framework to understand the interactions between hardware and software [30]. However, the tool remained useful to guide data analysis, and builds on growing work applying software conceptualisation of findings [30].

By analysing the software dimensions of interactions between health workers and camp authorities, our research demonstrated nuanced ways in which authoritarian forms of control can hinder care provision and potentially exacerbate the fear and stigma of health centres. For example, their perceptions of the Camp‐in‐Charge (CiC) were conflicted, as they felt support for patients was prioritised over theirs (health workers). Other qualitative research in the camps had also shown a favourable presence of authoritative control in health centres, due to increasing crime and violence in the camps [25]. However, our findings show this should be interpreted with caution, and previous social science research has argued that the consequences of significant top‐down, authoritative control in outbreak response encourage ‘disease exceptionalism’, [40] whereby community‐driven priorities and local health system governance structures are not considered [40, 41, 42]. It has also been found that strict authoritative control in outbreaks on communities and the local health response excludes community‐level participation in decision‐making [43, 44]. For example, while the involvement of the military in outbreak response activities in the 2014–16 Ebola epidemic in Sierra Leone was favoured by some frontline responders [45], an over‐reliance on authoritative control undermined community‐driven approaches to disease control [43, 44].

Despite the COVID‐19 response in the camps having relatively strong surveillance and IPC strategies to guide interventions, our findings, interpreted through a systems software perspective, suggest the presence of systemic barriers and weakening relationships between health workers and the health sector leadership team and management structures [16, 46]. For example, our research revealed significant coordination challenges, particularly with the health sector's leadership team's physical distance from the camps and failure to build rapport, described by participants as “sitting in cities”. The alarming accounts of health workers being discouraged by their organisations from wearing masks to suppress patient fears during the COVID‐19 pandemic reflected controlling behaviours and unequal favouring of the care needs of their patients. This was on top of the daily underlying fears of the rising security situations in the camps [47]. Mayhew et al. [44] (p.1744) state that for decision‐making power to be led by local people, humanitarian responses must “enable frontline responders (both local affected people and frontline health workers) to lead, shape, and be locally accountable for, their own crisis response”. To our knowledge, there is no sector‐wide technical guidance or system being used to promote inclusiveness in decision making for health workers or more widely in the global health cluster; however, global guidance can be used from WHO that recognises health workers as a priority stakeholder group in outbreak response. For example, WHO's COVID‐19 guidance for human resources for health managers and policymakers, encourages continuous transparent dialogue between health system leadership and frontline staff, and managers should have strong human resources for health information systems, including health workforce indicators to provide evidence‐based policies and decisions [48].

### 5.1 Implications for Policy and Practice

In the context of the Rohingya response in Cox's Bazar, our recommendations align with the current global “humanitarian reset” by reassessing how shifts in power and coordination mechanisms can improve health workers' working conditions, strengthen a locally driven humanitarian response, and subsequently improve quality of care and sustain a strong health workforce in crisis settings.

Poor resourcing and PPE shortages are common in crisis settings and should be seen as part of a broader global governance failure in outbreak responses that must be addressed by member states, global health leaders (such as in the UN), and responding organisations [5, 6, 9]. Relevant commitments are available in the 2025 World Health Assembly Resolution WHA78.16 on *Accelerating Action on the Global Health and Care Workforce by 2030,* which highlights the need to ensure adequate occupational safety standards and safe working conditions [3].

Evidence from COVID‐19 and Ebola responses in crisis settings shows that decentralising decision‐making and meaningful inclusion of local actors, including health workers, is essential [40, 41, 44]. Côté and Denis [49] argue that to address health workforce challenges, solutions should be co‐developed with health workers, rather than imposed by “organisational elites”. For example, in the Rohingya camps, where health workers remain highly vulnerable to infectious disease outbreaks, health sector strategies should aim to develop formal mechanisms for health workers' participation in decision‐making. This aligns with the 2025‐26 Joint Response Plan under health to better prepare for outbreaks [36](p38). To our knowledge, health sector feedback mechanisms do not exist within WHO's HSCT. Systematic engagement by WHO's health sector coordinator and health managers in health workers' daily work could improve patient satisfaction, quality of care, and preparedness for future outbreaks in the camps. For example, a health workforce working group could be established alongside other working groups to monitor their wellbeing and set up formal mechanisms for quality of care. This could include regular in‐camp consultation forums and anonymous feedback channels, which would require less travel and clinic disruption than hosting meetings in Cox's Bazar. Feedback could then be reported through WHO's HSCT's monthly meetings with health partners, attended by their health managers, and feedback from staff can be shared and recorded in their monthly bulletins.

To protect health workers' safety and strengthen resilience, governments and managing organisations must improve workforce policies and accountability mechanisms. The WHO Health Worker Safety Charter outlines key measures to address health worker safety, protection from violence, mental health support, and safeguards against physical risks and biological hazards [50]. Locally, organisations should implement duty of care protocols, such as adequate rest periods, psychosocial support access, and clear guidance for managing patient and community threats and violence, and working with camp authorities [48].

Finally, future health response research in the camps should be inclusive of health workers. For example, more research is needed on how participatory health systems software interventions might improve the quality of care and patient trust. Research approaches with health workers should also seek to foster safe ways to share information without risks to their employment.

## 6 Conclusion

Overall, by exploring the relationship between hardware and software elements in a health system during infectious disease outbreaks, our findings show that health workers' professional ecosystems must be better understood to build and sustain a strong, resilient healthcare workforce. With the global threats of infectious diseases, war, conflict, and climate change, access to quality healthcare and public health services for vulnerable populations is crucial in the host countries receiving refugees and migrants.

This study is the first to explore health workers' social and political environment in the Rohingya crisis; the insights provided are critical for improving infectious disease preparedness and response efforts, strengthening patient‐provider trust, and supporting mechanisms for health workers. Response efforts must be redesigned to include health workers' protection and safety, promote inclusiveness in outbreak decision‐making, and ensure preparedness practices are prioritised so that frontline health workers in Cox's Bazar are ready for future outbreaks they will inevitably face.

## Funding

This research is part of the PI's self‐funded PhD work and has received additional support through data collection grants from the LSHTM Travel Grant and the Queen Elizabeth II Platinum Jubilee Grant.

## Conflicts of Interest

The authors declare no conflicts of interest.

## Supporting information


Supporting Information S1

Supporting Information S2

## Data Availability

Research data are not shared to protect the privacy and confidentiality of participants in this study.
