# Supplementary material for: How Health Systems ‘Software’ Factors Affect Frontline Health Workers' Humanitarian Response Efforts During Infectious Disease Outbreaks in the Rohingya Refugee Camps, Cox's Bazar, Bangladesh
Source: Int J Health Plann Manage. 2026 May 14;41(4):740–52. doi: 10.1002/hpm.70088 (PMC13339748; doi:10.1002/hpm.70088)
Supplement: Supplementary file 1 — Supporting Information S1 [file HPM-41-740-s002.docx]

## Supplementary file 1: COREQ checklist

Consolidated criteria for reporting qualitative studies (COREQ): 32-item checklist

For manuscript: *“How health systems “software” factors affect frontline health workers’ care practices and response efforts during infectious disease outbreaks in the Rohingya refugee camps, Cox’s Bazar, Bangladesh”* by Venner et al.

Framework from: Tong A, Sainsbury P, Craig J. Consolidated criteria for reporting qualitative research (COREQ): a 32-item checklist for interviews and focus groups. International Journal for Quality in Health Care. 2007. Volume 19, Number 6: pp. 349 – 357

| **No** | **Item** | **Guide questions/description** | **Response** |
| --- | --- | --- | --- |
| **Domain 1: Research team and reflexivity** | | | |
| *Personal Characteristics* | | | |
| 1. | Interviewer/facilitator | Which author/s conducted the interview, focus group, or workshop? | The research team included two Bangladeshi and one Rohingya research assistants (two female, one male), while the PI was a foreign, white individual from North America and represented a Western institution, therefore may have influenced participant responses to either exaggerate or hide their challenges. The PI had extensive work experience in the camps before the research project, while this was predominantly seen as an advantage in terms of familiarity with the setting and healthcare workforce, it may have influenced biased interpretations of the data.  To mitigate bias, reflexivity of the interpretation of findings was practiced by conducting research team debriefs to triangulate perspectives of the data. In addition, data validation workshops and a presentation of preliminary findings to the health sector coordination group (in 2023), which were met with positive responses and opportunities for feedback, sought to mitigate these factors.  The Rohingya research assistant for this study was a part of debriefs on broad themes, but they weren’t present during the interviews or workshops with Bangladeshi HWs. This person also reviewed this paper for sense-checking. |
| 2. | Credentials | What were the researcher's credentials? *E.g. PhD, MD* | The interview team consisted of 1 PhD Candidate (LSHTM) with an MPH, two Bangladeshi research assistants had master's degree, and the third Rohingya research assistant had significant experience in conducting qualitative research and translation in the Rohingya camps.  Please see the author list for more information. |
| 3. | Occupation | What was their occupation at the time of the study? | The PI was a PhD Candidate, the others were research assistants. |
| 4. | Gender | Was the researcher male or female? | Two female Bangladeshi research assistants and one male Rohingya research assistant. One male translator was used for a workshop. |
| 5. | Experience and training | What experience or training did the researcher have? | The lead researcher had expertise in qualitative research and humanitarian programme management. The research assistants possessed extensive experience conducting research studies in the camps and providing translation services for humanitarian organisations in Cox’s Bazar. Additionally, all research assistants received training on data collection methods and research ethics specific to this study. |
| 6 | Partner details | Who are the research partners? | North South University, a university focusing on public health research in Dhaka, Bangladesh and with extensive research in the Rohingya refugee response in Cox’s Bazar. The London School of Hygiene and Tropical Medicine is a global public health academic institution. Friendship is a national Social Purpose Organisation (SPO) operating in Bangladesh with a strong health and research presence in the Rohingya camps and in crisis-affected areas of Bangladesh. The PI (GV) from LSHTM worked closely with NSU and Friendship to assist with data collection efforts and research support in the field. |
| *Relationship with participants* | | | |
| 6. | Relationship established | Was a relationship established prior to study commencement? | Participants were recruited through research partners’ assistance, word of mouth, the health sector database, and snowball sampling. For familiarisation with health organisations, the PI provided study information during the health sector coordination meetings before data collection to determine interest and ensure the researcher was known.  For the majority of participants, no prior formal relationship was established. Before data collection, senior members of participants' organisations were contacted to introduce the study, oriented on expectations, and to seek permission to engage with their health worker staff. All participants received a detailed introduction to the study and provided informed consent through discussions with the principal investigator (PI) and research assistants before data collection began.  Some participants had a prior professional relationship with the PI due to the PI’s previous, separate involvement in the camps with an NGO from 2018-2021.  For key informant interviews, all participants were contacted in advance by the PI and provided an overview of the research. |
| 7. | Participant knowledge of the interviewer | What did the participants know about the researcher? e*.g. personal goals, reasons for doing the research* | The general research objectives, along with the roles of the interviewer and note-taker, were clearly explained. |
| 8. | Interviewer characteristics | What characteristics were reported about the interviewer/facilitator? e.g. *Bias, assumptions, reasons and interests in the research topic* | Who the interviewer and assistants are, the PI’s experience working in the camps with health workers, the research assistants’ experience working in the camps, where they came from, how the interview will be conducted, and how data will be shared and reported with the protection mechanisms in place for the participant. |
| **Domain 2: study design** | | | |
| *Theoretical framework* | | | |
| 9. | Methodological orientation and Theory | What methodological orientation was stated to underpin the study? *e.g. grounded theory, discourse analysis, ethnography, phenomenology, content analysis* | This study employed an in-depth qualitative approach, with data analysed using thematic analysis and guided by a health systems analytical framework. We conducted an in-depth analysis using Braun and Clarke’s six-phase approach, applying both inductive and deductive methods. To strengthen theoretical coherence, we then drew on Sheikh et al.’s hardware–software framework to organise codes and map software-related themes, particularly those related to relationships and power, onto relevant system hardware. This hybrid use of thematic and framework analysis offered both flexibility and structure, enabling us to interpret health workers’ experiences across different levels of the humanitarian health system. |
| *Participant selection* | | | |
| 10. | Sampling | How were participants selected? *e.g. purposive, convenience, consecutive, snowball* | Participants were recruited with the assistance of research partners, through word of mouth, the health sector database, purposeful sampling amongst professional networks, snowball sampling, and by sharing study information with health organisations during the Health Sector Coordination Team (HSCT) meetings. Newly recruited health workers with minimal experience who did not have substance experience working in outbreak responses in the camps were excluded from the study . Efforts were made to achieve a balanced gender representation during recruitment.  Conducting research within this refugee setting posed challenges which included the navigation of complex power structures in the humanitarian sector and government authorities and contributed to delays in recruitment, data collection, and diversity of sample. |
| 11. | Method of approach | How were participants approached? e*.g. face-to-face, telephone, mail, email* | For in-depth interviews, focus groups, and workshops, some participants were recruited with the assistance of clinical managers. Managers were first contacted via email with an explanation of the study. If they expressed interest, they facilitated recruitment by identifying and connecting their organisations’ health workers to the research team.  Some participants were directly approached by email with the study information included. |
| 12. | Sample size | How many participants were in the study? | 33 in-depth interviews (IDIs) with frontline Health workers (clinical and public health) (Bangladeshi), four data validation workshops with 20 frontline clinical Health workers (clinical managers & MA in-charge, clinical officers, and 13 key informant interviews (KIIs) with senior health experts and humanitarian stakeholders.  Two participants from IDIs also participated in the workshops.  In total, 63 participants were in the study (after removal of the three duplicates).  Participant data with community health workers are not included in this paper. |
| 13. | Non-participation | How many people refused to participate or dropped out? Reasons? | At the end of their interview, one in-depth interview participant chose to drop out and their data was destroyed.  Two workshop participant dropped out unexpectedly due to work commitments, someone filled their spot with a similar role.  Seven KIIs serving as humanitarian stakeholders were approached with no response, reasons are unknown. |
| *Setting* | | | |
| 14. | Setting of data collection | Where was the data collected? e*.g. home, clinic, workplace* | In-depth interviews were conducted in private settings in Cox’s Bazar or Ukhiya towns, including designated meeting rooms at participants' organisational headquarters, private closed-door spaces, or clinic areas that were closed off. One interview was conducted on Zoom.  All four data validation workshops were held in a private meeting space in Cox’s Bazar and lasted four hours with breaks and refreshments.  Workshops 1 and 2 were held in English while workshops 3 and 4 were held in Bengali and were co-led with the Research Assistant speaking Bengali and presenting findings to participants and translating with the PI where natural breaks in topics occurred. Another Bengali-speaking RA or translator was present, taking research notes and also assisting with translation for the PI.  The location of KIIs included private meeting spaces in Cox’s Bazar, their private office spaces, or remotely on Zoom. |
| 15. | Presence of non-participants | Was anyone else present besides the participants and researchers? | No one was present during IDIs, workshops, or KIIs. For interviews held in private clinic spaces, occasionally a clinical worker would approach the room for a question to the participant in need of professional assistance, the interview and recording would stop until the colleague left. |
| 16. | Description of sample | What are the important characteristics of the sample? *e.g. demographic data, date* | Both genders (male and female) with formal education in health care and with at least 6 months experience working in the camps.  The duration of data collection was between October 2022-December 2023 with two field visits. |
| *Data collection* | | | |
| 17. | Interview guide | Were questions, prompts, guides provided by the authors? Was it pilot-tested? | Semi-structured interview topic guides for IDIs centred around their journey working in the camps, experiences of infectious disease outbreaks in the camps, training and professional development services, camp environment and safety, relationships with patients and communities, and recommendations for future support*.* The topic guide was pilot-tested and subsequently modified.  Topic guides for KIIs centred around their roles and decision-making experiences responding to infectious disease responses in the camps, comparing outbreak responses, challenges and successes in outbreak response, training and support structures for Health workers, and recommendations. The topic guide was pilot-tested and subsequently modified.  Probing experiences from the COVID-19 pandemic were applied due to it being present during the time of data collection and having a greater impact on their work. |
| 18. | Repeat interviews | Were repeat interviews carried out? If yes, how many? | No repeat interviews were carried out.  Two participants from IDIs also participated in the data validation workshops. |
| 19. | Audio/visual recording | Did the research use audio or visual recording to collect the data? | Audio recordings were carried out with those who consented.  Audio recording for IDIs, workshops, and KIIs was used with password-protected audio devices and stored with a unique, non-identifiable ID. Interviews were conducted remotely used Zoom audio recording.  For interviewees that did not want to be recorded, detailed field notes were taken.  The PI (GV) and research assistants (TA and MDM) transcribed IDI transcripts conducted in English. Bengali interviews were processed in two stages: first, RAs transcribed the audio recordings using the real-time English translation provided during the interview; second, the PI and RAs reviewed these transcripts and selected key sections for verbatim transcription from the original Bengali.  Data validation workshops were fully translated and transcribed, while introductory explanations of the study were omitted because both English and Bengali versions were already available. All quotes included in this paper were transcribed verbatim, and all identifiable information was removed prior to analysis.  All KIIs were in English.Participants' information was stored in LSHTM’s encrypted folder and will be stored for 10 years. |
| 20. | Field notes | Were field notes made during and/or after the interview or focus group? | Field notes by hand or on computer were made during the interviews, workshops, and KIIs by all interviewers and translators present. Field notes were then typed up and sent to the PI. Field notes were given a participant ID and stored with their respective audio file and transcript. |
| 21. | Duration | What was the duration of the interviews or focus group? | IDIs and KIIs lasted between 30 minutes and 1.5 hours.  Data validation workshops lasted 4 hours with breaks. |
| 22. | Data saturation | Was data saturation discussed? | Data saturation was discussed with the research team and then verified with a member of the senior research team (SM). |
| 23. | Transcripts returned | Were transcripts returned to participants for comment and/or correction? | No. |
| **Domain 3: analysis and findings** | | | |
| Data analysis | | | |
| 24. | Number of data coders | How many data coders coded the data? | The PI (GV) was the only data coder after scripts were transcribed. Codes were periodically discussed with SM and JP for validity. |
| 25. | Description of the coding tree | Did authors provide a description of the coding tree? | Yes, a codebook was developed with overarching themes and sub-themes surrounding governance and authority; health sector coordination; and organisational management. This is reflected in the findings. |
| 26. | Derivation of themes | Were themes identified in advance or derived from the data? | Using the topic guide and research questions, preliminary findings and emerging themes from the first data collection visit were initially mapped by members of the field team (GV and TA). GV then conducted a rapid thematic analysis of approximately half of the IDI transcripts to generate early findings and group emerging themes for the data validation workshops.  After the workshops and completion of the second data collection visit, the field team (GV, TA, MDM, MMZ) revisited and refined the thematic map. GV subsequently undertook an in-depth analysis using Braun and Clarke’s six-phase approach, applying both inductive and deductive methods. Themes were shaped by the workshop discussions and topic guides, while inductive coding allowed for the identification of additional nuances. Research notes were coded for interviews that were not audio recorded.  To strengthen theoretical coherence, Sheikh et al.’s hardware–software health systems framework was applied deductively. This framework guided the organisation of codes and the mapping of software-related themes, particularly those relating to feeling valued, motivation, relationships, feeling safe and prepared, mental health and wellbeing, and power, onto relevant hardware components of the system, including humanitarian response governance, health sector coordination leadership, and organisational management. The results were organised according to levels of response governance structure (hardware), within which software-related themes were examined. The PI developed the coding framework and sense-checked it with research assistants (TA, MM, MN), and SM and JP which consisted of frequent team debriefings of themes. Research partners (MU and AH) were also consulted on overall themes and approach of the analysis.  This hybrid approach, combining thematic analysis with framework analysis, provided the flexibility needed to capture the detailed experiences of health workers and the structure necessary to interpret these experiences across different levels of the humanitarian health system.  Theoretical saturation was reached after detailed coding in NVivo12 of 22 of the 33 IDIs and two of the four data validation workshops. A rapid analysis of the remaining 11 IDIs and two workshops was conducted manually to ensure consistency and identify any deviations from the main themes. All KIIs were conducted with higher-level humanitarian personnel and were rapidly analysed to assess whether they supported or challenged the emerging thematic structure. |
| 27. | Software | What software, if applicable, was used to manage the data? | NVivo12. |
| 28. | Participant checking | Did participants provide feedback on the findings? | Themes from the IDIs were presented in all of the data validation workshops to confirm, refute, and explore new themes. |
| Reporting | | | |
| 29. | Quotations presented | Were participant quotations presented to illustrate the themes / findings? Was each quotation identified? e*.g. participant number* | Yes, quotes were given a participant ID that protects anonymity. |
| 30. | Data and findings consistent | Was there consistency between the data presented and the findings? | Yes. |
| 31. | Clarity of major themes | Were major themes clearly presented in the findings? | Yes. |
| 32. | Clarity of minor themes | Is there a description of diverse cases or discussion of minor themes? | Some deviations were highlighted in the findings to contrast dominate themes and findings. |
