# Supplementary material for: How Health Systems ‘Software’ Factors Affect Frontline Health Workers' Humanitarian Response Efforts During Infectious Disease Outbreaks in the Rohingya Refugee Camps, Cox's Bazar, Bangladesh
Source: Int J Health Plann Manage. 2026 May 14;41(4):740–52. doi: 10.1002/hpm.70088 (PMC13339748; doi:10.1002/hpm.70088)
Supplement: Supplementary file 2 — Supporting Information S2 [file HPM-41-740-s001.docx]

## Supplementary file 2: Professional cadres and detailed summaries of interview participants

Table 1: Professional cadre definitions

| **Medical Officer/Clinical Officer**  **Clinical Manager/Clinical Supervisor** | Medical professionals with formal medical training from a recognised university with MBBS with BDMC Registration  Medical officers (as above) in charge of the clinic activities and staff. |
| --- | --- |
| **Medical Assistant**  **Medical Assistant In-Charge** | A medical professional with formal training (a diploma) from a recognised Medical Assistant Training School (MATs) or Institute and registered with State Medical Faculty of Bangladesh (SMF) registration.  A medical assistant (as above) in charge of medical assistants in the clinic, sometimes they are responsible for the whole clinic if it is a health post. |
| **Nurse** | A nurse professional with formal training and a recognised diploma or certificate from a nursing and midwifery institution and registered with the Bangladesh Nursing and Midwifery Council BNMC) |
| **Midwife** | A midwife professional with formal training and a recognised diploma or certificate from a nursing and midwifery institution and registered with the Bangladesh Nursing and Midwifery Council BNMC) |

Table 3: Summary of in-depth interviews

| **Data collection type** | **Number of interviews** | **Background** | **Gender** | **Professional background** | **Type of health organisations** |
| --- | --- | --- | --- | --- | --- |
| **In-depth Interviews (IDIs)** | 33 | Bangladeshi | 13 females  20 males | 7 clinical officers /clinical managers (trained medical doctors)  5 clinical officers (trained medical doctors)  10 medical assistants (MA)/MA supervisors  3 nurses/nurse supervisors  4 midwives  2 health promoters/IPC experts  1 mental health manager  1 dental assistant | 3 Bangladeshi NGOs  4 INGOs |

Table 4: Summary of data validation workshops with clinical health workers

| **Data collection type** | **Number of interviews** | **Background** | **Gender** | **Professional background** | **Type of health organisations** |
| --- | --- | --- | --- | --- | --- |
| **Data validation workshops (WS)**  (20 participants total, 2 repeated participants from IDIs) | WS01 | 5 Bangladeshi participants | 2 females  3 males | 4 clinical managers  1 MA in-charge | 4 Bangladeshi NGOs  5 INGOS |
|  | WS02 | 5 Bangladeshi participants | 1 female  4 males | All clinical officers |  |
|  | WS03 | 5 Bangladeshi participants | 1 female  4 males | All MAs |  |
|  | WS04 | 5 Bangladeshi participants | 5 females | 3 nurses  2 midwives |  |

Table 5 Summary of key informant interviews with humanitarian stakeholders

| **Data collection type** | **Number of interviews** | **Background** | **Gender** | **Professional background** | **Type of health organisations** |
| --- | --- | --- | --- | --- | --- |
| **Key informant interviews (KIIs)** | 13 | 8 International    5 Bangladeshi | 5 females  8 males | Humanitarian and technical experts, advisors, decision-makers, and/or funders with roles or influence in the health response (specific backgrounds not given to protect anonymity) | 7 INGOs and Inter-Governmental organisations  1 Bangladeshi NGO  1 Funder |
